# Serum Albumin Redox States: More Than Oxidative Stress Biomarker

**DOI:** 10.3390/antiox10040503

**Published:** 2021-03-24

**Authors:** Fuka Tabata, Yasuaki Wada, Satomi Kawakami, Kazuhiro Miyaji

**Affiliations:** Wellness & Nutrition Science Institute, Morinaga Milk Industry Co., Ltd., 5-1-83 Higashihara, Zama, Kanagawa 252-8583, Japan; fuuka-tabata131@morinagamilk.co.jp (F.T.); s_kawakm@morinagamilk.co.jp (S.K.); k_miyazi@morinagamilk.co.jp (K.M.)

**Keywords:** albumin, biomarker, oxidative stress, serum albumin redox state

## Abstract

Serum albumin is the most abundant circulating protein in mammals including humans. It has three isoforms according to the redox state of the free cysteine residue at position 34, named as mercaptalbumin (reduced albumin), non-mercaptalbumin-1 and -2 (oxidized albumin), respectively. The serum albumin redox state has long been viewed as a biomarker of systemic oxidative stress, as the redox state shifts to a more oxidized state in response to the severity of the pathological condition in various diseases such as liver diseases and renal failures. However, recent ex vivo studies revealed oxidized albumin per se could aggravate the pathological conditions. Furthermore, the possibility of the serum albumin redox state as a sensitive protein nutrition biomarker has also been demonstrated in a series of animal studies. A paradigm shift is thus ongoing in the research field of the serum albumin. This article provides an updated overview of analytical techniques for serum albumin redox state and its association with human health, focusing on recent findings.

## 1. Introduction

**Figure 1:**
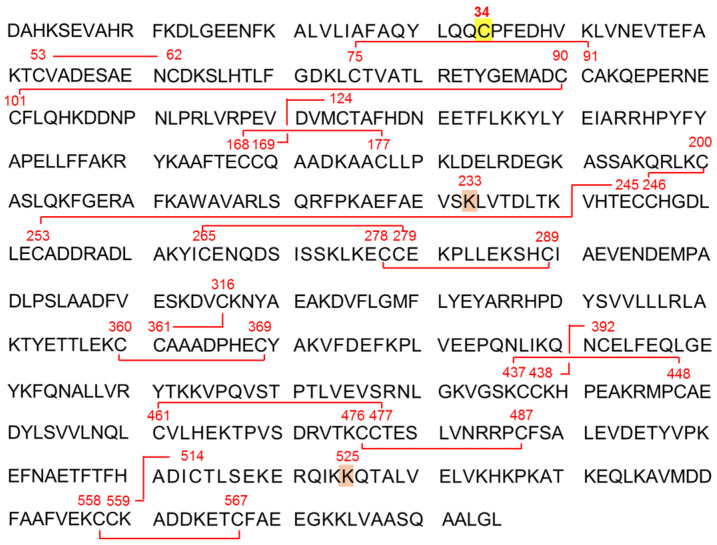
Amino acid sequence of human serum albumin (ALB). Human serum ALB, which is presented in the single-letter amino acid code, consists of a polypeptide chain of 585 amino acid residues including 35 cysteine (Cys) residues. Thirty-four of Cys residues form disulfide bridges, while the remaining single Cys residue at position 34 is free and redox-active. Cysteinylation, homocysteinylation, sulfinylation on Cys34 and glycosylation on lysine residue at position 233 (Lys233) and Lys525 have been confirmed [9].

Albumin (ALB) is the most abundant protein in serum, which has a molecular weight of approx. 66 kDa and is normally present at 35–45 g/L (approx. 0.6 mM) [1]. It is exclusively synthesized in the liver, before being secreted into the circulation [2]. Human serum ALB consists of a single polypeptide chain of 585 amino acid residues, and has a total of 35 cysteine (Cys) residues. The 34 Cys residues are involved in formation of 17 intramolecular disulfide bridges, contributing to its heart-shaped tertiary structure (Figure 1). The remaining single Cys residue at position 34 (Cys34) is free and redox-active. The primary role of ALB in the circulation is to provide colloidal osmotic pressure. As ALB is quite abundant in serum and relatively lower molecular weight, this protein contributes to approx. 80% of total colloidal osmotic pressure [3]. Conversely, ALB synthesis in liver is responsive to colloidal osmotic pressure; hepatic ALB gene expression receives a feedback regulation by colloidal osmotic pressure, thereby maintaining its homeostasis [4,5]. Collectively, serum ALB is thus a key molecule for the homeostasis of colloidal osmotic pressure. Another important role of serum ALB is to carry various kinds of endogenous and exogenous ligands. The ligands include endogenous compounds such as long-chain fatty acids, bilirubin, metal ions (zinc, copper, calcium, etc.), and exogenous drug substances such as warfarin and ibuprofen [6]. The presence of multiple ligand binding pockets and its long half-life in the circulation (approx. 15 days) makes this protein as an attractive vehicle for novel drug delivery systems. Furthermore, serum ALB is involved in the redox homeostasis in the circulation [7]. Whereas small thiols Cys and glutathione (GSH) are the major antioxidants contributing to intracellular redox homeostasis, the most abundant thiol in plasma is the Cys34 residue in ALB, which exerts anti-oxidative activity and circumvent systemic oxidative stress. The Cys34 residue scavenges multiple reactive oxygen and nitrogen species such as hydrogen peroxide (H_2_O_2_), peroxynitrite (ONOO^−^), superoxide (O_2_^−^), and hypochlorous acid (HOCl) [7,8]. Lastly, serum ALB level has been used classically as a biomarker of protein nutritional status, with the level < 35 g/L defined as hypoalbuminemia, but low serum ALB level is currently viewed more as a risk factor and a predictor of morbidity/mortality regardless of the implicated diseases [1].

**Figure 2:**
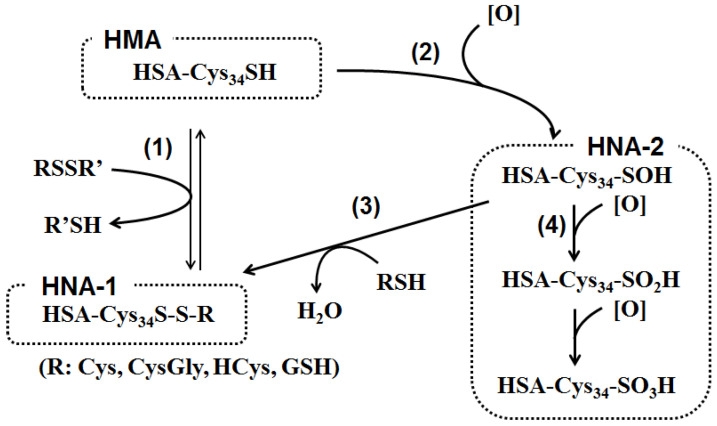
Pathway for human serum albumin (ALB) redox state. (1) The free thiol group in cysteine residue at position 34 (Cys34) of human serum mercaptalbumin (HMA) receives a disulfide exchange reaction with low molecular weight disulfides consisting of cysteine (Cys), cysteinylglycine (CysGly), homocysteine (HCys) and/or glutathione (GSH), to form a mixed disulfide on Cys34. The resulting oxidized ALB is designated as human non-mercaptalbumin-1 (HNA-1). (2) Alternatively, the Cys34 reacts with several reactive oxygen and nitrogen species such as hydrogen peroxide (H_2_O_2_), peroxynitrite (ONOO^−^), superoxide (O_2_^−^), or hypochlorous acid (HOCl), and is initially oxidized to sulfenic acid. (3) Sulfenic acid reacts with cysteine and other thiols to form HNA-1. (4) On the other hand, sulfenic acid is further oxidized by reactive oxygen species to sulfinic and sulfonic acid. These oxidized ALB isoforms are designated as HNA-2.

The thiol group of Cys34 residue is redox-active as described above, and the redox state grants the heterogeneity of serum ALB isoforms. They can be separated chromatographically: (i) the free thiol form called human mercaptalbumin (HMA); (ii) a mixed disulfide with a small thiol compound such as Cys and cysteinylglycine (CysGly), as well as homocysteine (HCys) and GSH albeit to a lesser extent, called human non-mercaptalbumin1 (HNA-1); and (iii) the higher oxidized form of cysteine residue as sulfinic or sulfonic acid, called HNA-2 (2) (Figure 2). In healthy young subjects, HMA accounts for 70–80%, HNA-1 for 20–30%, and HNA-2 for 2–5% of total ALB, respectively [10]. Reduced and oxidized forms of ALB are different in physical properties. Colloidal osmotic pressure was increased by ALB oxidation in vitro using HOCl, and colloidal osmotic pressure was higher than predicted from serum ALB levels in chronic kidney disease patients [3]. Binding affinities for endogenous ligands, bilirubin and tryptophan, as well as exogenous drug substances, warfarin and diazepam, are decreased in proportion to ALB oxidation (cysteinylation on Cys34) [11]. Binding affinities for lipid mediators also differs between reduced and oxidized serum ALB isoforms; proatherosclerotic lipids such as lysophosphatidylcholine and lysophosphatidic acid have higher affinities for oxidized ALB isoform, while anti-atherosclerotic mediators derived from eicosapentaenoic acid and docosahexaenoic acid possess higher affinities for reduced ALB isoform [12]. Furthermore, ALB oxidation (the increased HNA-1 level) was accompanied by decreased anti-oxidative activity as measured by a total radical-trapping antioxidant parameter assay [13]. The oxidation of biological components such as proteins, lipids, carbohydrates and DNA proceeds when anti-oxidative potential is attenuated. This leads to impaired intracellular signaling, cellular dysfunction, etc., ultimately contributing to the pathogenesis of various kinds of diseases including liver diseases, renal failure, diabetes mellitus, atherosclerosis, cardiovascular diseases, cancer and infertility [14]. Serum ALB redox state has extensively been investigated in the association with various kinds of diseases and some kinds of physiological conditions. Increases in oxidized ALB have been observed in patients with liver disease, renal failures, diabetes mellitus, and hypertension, in subjects after strenuous exercise, and in aged population [11,15,16,17,18]; all of these have exclusively been viewed as the manifestation of oxidative stress. However, several recent ex vivo studies have indicated that oxidized ALB per se could aggravate these pathological conditions [15,19,20,21,22,23,24]. Furthermore, it has recently become evident that ALB redox state can be a novel biomarker that sensitively reflects protein nutritional status [25,26]. Thus, a paradigm shift is ongoing in the research field of serum ALB.

The article provides an update of the studies on serum ALB redox state. Recent advances in analytical chemistry of ALB redox state are also discussed.

## 2. Analytical Chemistry of Serum ALB Redox State

ALB isoforms have been conventionally separated by high-performance liquid chromatography (HPLC) systems and detected spectrometrically or colorimetrically. The recent advent of high-resolution mass spectrometry (MS) technology has made it possible to characterize the chemical structures of post-translational modifications in oxidized ALB. For clinical settings that are not equipped with these instruments, colorimetric assays have been proposed for determining serum ALB redox state.

### 2.1. HPLC Analysis

Era et al. established a basis for HPLC analysis of serum ALB redox state in humans [27], which was then revised by Hayashi et al. [28]. It adopts an anion-exchange column having diethylaminoethyl (DEAE) as a functional group with a 40 min gradient of increasing EtOH concentration in the solvent of 0.4 M Na_2_SO_4_ and 0.05 M CH_3_COONa (pH 4.85). This system has also been modified to separate rat ALB isoforms by modulating the gradient condition, although the separation of NA-1 and NA-2 is less evident in rats compared with human counterparts [29]. Recently, Yasuoka et al. developed a rapid HPLC system that enables the separation ALB isoforms in 9 min [30,31,32]. This system adopts an in-house column of anion-exchange gel prepared from polyvinyl alcohol cross-linked gel reacted with diethylamine, with two eluents of 25 mM phosphoric acid buffer containing 60 mM sodium sulfate (pH 6.0) and 250 mM magnesium chloride solution. Still, it may be kept in mind that this system focuses on the separation of HMA from HNAs, and the separation of HNA-1 and HNA-2 is less evident compared with the system by Hayashi et al. [28].

For the detection of serum ALB isoforms in HPLC systems, the fluorescence emission of a tryptophan residue at position 214 in human serum ALB (340 nm for emission and 280 nm for excitation) is more frequently used compared with measurement of UV absorbance at 215 nm derived from peptide bonds in the protein. The UV absorbance at 215 nm involves an interference with the separation of HMA and HNA-1 by uric acid [33], which is a critical obstacle especially for the determination of serum ALB redox state in chronic kidney disease (CKD) patients [34]. Ueyama et al. proposed an HPLC system for addressing this issue [33]; the method employed a post-column derivatization in which serum ALB is reacted with bromocresol green (BCG), thereby circumventing the interference by uric acid. On the other hand, detection by fluorescence emission is also accompanied by a drawback in the case of advanced liver disease patients. Namely, an increase in serum bilirubin level in these patients causes an increase in bilirubin-bound HMA, which would attenuate fluorescence emission by HMA [35]. Other methods for determining serum albumin redox state include quantification of thiol group by Ellman’s assay using 5,5-dithiobis-2-nitrobenzoic acid (DTNB) [36], and measurement of carbonylated protein using 2,4-dinitrophenylhydrazine (DNPH) [37].

Some improvements have been made on HPLC systems for serum ALB redox state analysis. However, while the use of HPLC systems are enough to determine the ratios of reduced and oxidized serum ALB isoforms, they are not able to characterize chemical structures of the post-translational modifications in oxidized ALB isoforms.

### 2.2. MS Analysis of Intact ALB

The use of MS techniques has been applied to the characterization of post-translational modifications in serum ALB. One of the most common methods for analyzing serum ALB is to analyze ALB in intact forms and observe the “mass shift” between the reduced and oxidized ALB isoforms, there by deducing the post-translational modifications. Matrix-assisted laser desorption/ionization time-of-flight mass spectrometry (MALDI-TOF MS) is a typical method for analyzing intact proteins, where proteins are ionized mainly as positively and singly charged ions. Specifically, the application of MALDI-TOF-MS analysis to serum ALB appears to be limited presumably due to its insufficient resolution. Nakashima et al. examined serum ALB redox state in hyperlipidemic patients using MALDI-TOF-MS [9]. It was found that the molecular mass of oxidized ALB was increased by approx. 200 Da in comparison with reduced ALB, implicating post-translational modifications of serum ALB by low molecular weight compounds. However, the mass shift was not accurate enough to deduce the chemical structure, and the authors used another MS technique to complement it. Compared with MALDI-TOF-MS, electrospray ionization-TOF-MS in combination with LC (LC-ESI-TOF-MS) is more frequently selected for serum ALB analysis. Spectra derived from ALB are observed as a series of multiply charged positive ions with the charge state ranges depending on experimental conditions. Typically, serum ALB is detected as spectra with charge states +15–60 (i.e., with *m*/*z* 1000–4000), and “deconvolution” of the spectra is executed in order to obtain the molecular weight. Judging from the mass shifts from the reduced ALB, post-translational modifications in serum ALB have been deduced as N-terminal loss of asparagil-alanine residue (−186), C-terminal of leucine residue (−113), cysteinylation (+119), homocysteinylation (+133), glycation (+162), and sulfinylation/sulfonylation (+32/+48) [21,38,39,40,41,42,43,44,45]. However, the “observed mass” of reduced ALB has been frequently different from its “theoretical mass (66,438 kDa)” by approx. 1–5 Da, despite the fact that this observed mass needs to be used as a reference for calculating mass shifts. Besides, chemical structures of post-translational modifications in serum ALB have been deduced even though their observed mass shifts were found to be different from the theoretical ones by 1–2 Da. Thus, these deviations considerably cast doubt on the accuracy of post-translational modifications of serum ALB estimated by this MS technique. Furthermore, this MS technique cannot indicate the positions of post-translational modifications in serum ALB, warranting alternative techniques that compensate for these drawbacks.

Bottom-up proteomics is a method that enables site-specific identification of post-translational modifications in proteins. Protein samples are digested using sequence-grade digestive enzymes such as trypsin and serine protease Glu-C, and the resulting peptides are applied to LC-MS/MS analysis. Spectral data obtained are then analyzed in a database-dependent manner in order to identify amino acid sequences of the resulting peptides, post-translational modifications in the amino acid sequences, and the positions of post-translational modifications in the sequences [46]. In this technique, spectra of peptides of ~5–20 amino acid residues are generally detected with charge state +2–4, and assignment of the spectra to peptide sequence are made with the match tolerance typically set to ~10 ppm. This makes it possible to identify site-specific identification of post-translational modifications in proteins of interest with high credibility. However, this MS technique has been less used in comparison with the analysis of intact serum ALB. It may be because bottom-up proteomics normally employs reductions of disulfide bonds in proteins, followed by alkylation of thiol residues, for decomposing the tertiary structures and improving digestion efficiency. These procedures are not applicable to serum ALB redox state; a mixed disulfide on Cys34 with low molecular weight thiols is one of the major targets of serum ALB modifications, and a treatment with a reducing agent is not compatible with the mixed disulfide analysis. Some studies performed enzymatic digestion of serum ALB under non-reducing condition simply in the same procedure as reducing condition but devoid of a reducing agent [39,45], while Nakashima et al. adopted a protease enhancer for improving enzymatic digestion efficiency under non-reducing condition [9]. Cysteinylation, homocysteinylation, sulfinylation on Cys34, and glycation on a lysine residue at position 233 (Lys233) and Lys525 have been observed in these bottom-up proteomics (Figure 1). Notably, the study by Nakashima et al. reported cysteinylation and homocysteinylation on Cys residues other than Cys34 in serum ALB [9], despite the fact that these Cys residues are considered involved in the formation of intramolecular disulfide bridges [2]. The authors speculated that disulfide bond shuffling could take place in Cys residues other than Cys34 through thiol/disulfide interchange reactions in human serum ALB, as observed in the bovine counterpart [47], and the Cys residues normally involved in disulfide bridges might react with small thiol compounds. On the other hand, Grigoryan et al. dared the standard proteomic analysis (i.e., using a reducing agent) on serum ALB, focusing on sulfinylation/sulfonylation, and ignoring the mixed disulfide on Cys34 [48]. They confirmed sulfinylation and sulfonylation on Cys34, and even unraveled a mass shift corresponding to “addition of one oxygen atom and loss of two hydrogen atoms”, which was deduced as a sulfinamide formed by intramolecular reaction between Cys34 and the adjacent glutamine at position 33.

Bottom-up proteomics of serum ALB is thus quite informative, and could be more widely used in the field of ALB research. Still, when considering the incompatibility of the use of reducing agents with the analyses of mixed disulfides on Cys34, the addition of other analytical methods that complement bottom-up proteomics would be more desirable. Brioschi et al. analyzed small thiol compounds originally bound to serum ALB as mixed disulfides [13]. These compounds were released by treating serum ALB with a reducing agent tris(2-carboxyethyl)phosphine, and they were then derivatized with 4-[2-(dimethylamino)ethylaminosulfonyl]-7-chloro-2,1,3-benzoxadiazole (DAABD-Cl). This derivatization provides positively charged ions that are sensitively detected in LC-MS analysis, as well as gives a specific fragment ion (*m*/*z* = 72.2) in the parental ion scan mode [49]. These authors substantiated cysteinylation and homocysteinylation in oxidized serum ALB, which was initially confirmed by bottom-up proteomics. It may be noted, however, that these bottom-up proteomics analyze enzymatically digested serum ALB, and they may be less efficient for determine the ratios of reduced and oxidized serum ALB isoforms.

### 2.3. In Vitro Preparation of Model Oxidized Forms of Serum ALB

Chemical reactions involved in the formation of oxidized forms of serum ALB have been well documented by several literatures [50,51] (Figure 1). For the formation of HNA-1, i.e., mixed disulfides between Cys34 in serum ALB and lower molecular weight thiols, two reaction pathways have been postulated. The first one is the disulfide exchange reaction between the thiol residues of Cys34 in HMA and low molecular weight disulfides consisting of Cys, CysGly, HCys or GSH, to form a mixed disulfide on Cys34. The second one is mediated by oxidation of Cys34 to sulfenic acid by reactive oxygen species such as H_2_O_2_, ONOO^−^, O_2_^−^, and HOCl, and the resulting sulfenic acid is subsequently reacted with a low molecular weight free thiol to form a mixed disulfide. Sulfinic acid is alternatively oxidized further to sulfinic or sulfonic acid, i.e., the generation of HNA-2.

Taking these pathways into consideration, several research groups attempted the chemical generation of these serum ALB isoforms as experimental standards for HPLC and LC-MS analyses. Era et al. examined these as early as in 1988 [27]; they treated commercially obtained human serum ALB with cystine or glutathione disulfide (GSSG), and confirmed an increase in HNA-1 level by HPLC. They also treated GSH to human serum ALB, and found an increase in HMA level, indicating a reverse/reducing reaction where a mix disulfide on Cys34 of HNA-1 was converted to a free thiol residue (formation of HMA). This research group conducted similar experiments in rat serum ALB [29]; Hayashi et al. treated serum ALB with GSH, GSSG, and peroxide (H_2_O_2_), and demonstrated increases in MA, NA-1, NA-2 levels, respectively. Recent studies also demonstrated these chemical reactions, and the resulting ALB isoforms were analyzed by LC-MS. Leblanc et al. treated human serum ALB with CysGly and H_2_O_2_, and indicated increased levels of a mixed disulfide with CysGly and sulfinylation on Cys34 [45]. Ohashi et al. treated commercially obtained human serum ALB with cysteine and showed an increase in HMA level [52]. Notably, the increased HMA level was maintained as long as for 24 h when the low molecular weight fraction was removed by dialysis before the incubation, whereas the HMA level decreased and HNA-1 level increased again when the incubation was made without dialysis. Thus, serum ALB redox state is susceptible to oxidation by low molecular weight thiols surrounding serum ALB after blood has been drawn.

### 2.4. Stabilization of Serum ALB Redox State

Measurement of serum ALB redox is of biological significance in the context of various pathological and physiological conditions, as is discussed later in this review. However, serum ALB redox state is highly susceptible to storage conditions. Namely. serum ALB redox state is stable so long as serum samples are stored at approx. −80 °C, but ALB oxidation proceeds spontaneously at >−30 °C and the oxidation intensifies as the storage temperature increases [10]. Low molecular weight compounds surrounding serum ALB are likely responsible for the instability as described above [52]. This is a serious problem for securing the credibility of ALB redox state analysis. Kubota et al. investigated the optimized condition for stabilizing ALB redox state in heparin plasma obtained from healthy volunteers [53]. It was found that ALB redox state was stable at 10 °C at least for 24 h when plasma was diluted to ≥50-fold with 50 mM sodium phosphate buffer (pH 6.0), an eluent buffer for HPLC analysis. The stability of plasma ALB redox state was also confirmed when it was diluted to 100-fold with 50 mM sodium phosphate buffer with the range of pH 4.0–9.0; ALB redox state was stable for as long as 48 h when the pH was adjusted to 4.0–7.0. These authors also found that the use of sodium citrate as an anti-coagulant, but not heparin, contributed to the stability of plasma ALB redox state. Namely, addition of 0.5 M sodium citrate to whole blood in a ratio of 1:9 stabilized the plasma ALB redox state for as long as 72 h when stored in the form of whole blood at 4 °C. This observation was then supported by a study by Yasukawa et al. [31]. Namely, among the five kinds of anti-coagulants tested, the use of sodium citrate was the most effective to the prevention of plasma ALB oxidation at 4 °C. Sodium citrate was especially effective when it was added in a final concentration ≥70 mM with the pH 5.0–6.0. Collectively, serum/plasma ALB redox state can be stabilized by diluting the samples with appropriate buffers with appropriate dilution factors, and/or the use of sodium citrate as an anticoagulant for obtaining plasma.

### 2.5. Colorimetric Determination of Serum ALB Redox State

Two kinds of colorimetric methods have been commonly used, i.e., the bromocresol purple (BCP) method and the BCG method for measurement of serum ALB concentration [54]. The BCP method has been less used compared with the BCG method, as the binding affinities are different between reduced and oxidized serum ALB isoforms, leading to considerable uncertainties and biases in serum ALB level determination. Muramoto et al. developed a modified BCP method [55], where the difference in binding affinities was resolved through facilitating conversion of reduced ALB to oxidized ALB by adding sodium dodecyl sulfate and 5,5′-dithiobis(2-nitrobenzoic acid) to the BCP reagent. A recent study by Yoshihiro et al. is notable as they applied the modified BCP method to the determination of serum ALB redox state [56]. Namely, serum samples are reacted with BCP both in the presence and absence of sodium dodecyl sulfate and 5,5′-dithiobis(2-nitrobenzoic acid) under a condition where BCP sensitively reacts with oxidized ALB. Serum ALB redox state is expressed by the ratio of OD_600_ in the presence and absence of the above reagents. On the other hand, the BCG method also has several defectives. Namely, BCG cross-reacts with other serum proteins such as globulin [54], which could cause the overestimation of serum ALB levels. Besides, the binding affinities of BCG to serum ALB is attenuated when ALB is carbonylated or glycoxidized [57], which would also hamper the accurate determination of serum ALB level in some types of patients. Still, Michelis et al. leveraged the latter shortcoming of BCG, and developed a colorimetric method for determining the degree of ALB oxidation, termed “ALB detection index” [57]. This index is expressed as the ratio of serum ALB level determined by BCG to the one determined by OD_280_ or ELISA. The ALB detection index has been adopted in some clinical studies [3,19,22]. Although these colorimetric methods are less accurate and informative in quantification compared with HPLC and LC-MS methods, they are simple, rapid, and sufficiently reliable in many clinical settings.

Characteristics of colorimetric methods are compared with those of HPLC and LC-MS methods, which is summarized in Table 1.

## 3. Serum ALB Redox State in Pathological Conditions

As mentioned above, serum ALB redox state has extensively been focused on in the context of oxidative stress induced by various diseases such as liver diseases, renal failures, diabetes mellitus, etc. Recent clinical studies have further substantiated the relationship between serum ALB redox state and the severity of these diseases. Some of these studies have even elucidated that increased oxidized serum ALB in the diseases could be involved in the progression of the symptoms. Serum ALB redox state in pathological conditions is summarized in Table 2.

### 3.1. Liver Diseases

The relationship between liver diseases and serum ALB redox state has well been documented for the last few decades. Serum ALB redox state was shifted to a more oxidized state in patients with liver cirrhosis [11,21,23,43,58], and acute-on-chronic liver failure (ACLF) [35,59,60,61]. ALB oxidation increased as the pathological severity progressed [11,21,35,58,61], which has been viewed as a manifestation of oxidative stress caused by excessive iron storage or hepatosteatosis. An increase in the HNA-1 level was generally observed [11,21,23,35,43,58,60,61,62,63], whereas the increased level of HNA-2 level was also found [21,23,35,59,61,63], especially in patients with ACLF [59,61]. Branched-chain amino acids (BCAAs) are provided to cirrhotic patients for relieving the hypoalbuminemia [64], and the oral BCAA supplementation has also alleviated the oxidized shift of serum ALB [58,62]. This can be interpreted as meanings that 1) BCAAs are deficient in liver patients and the supplementation would contribute to an increase in the substances for ALB synthesis, and 2) de novo ALB synthesis (i.e., influx of reduced ALB) would be increased by BCAA supplementation through the stimulation mammalian target rapamycin (mTOR) signaling pathway [2]. Reversal of serum ALB redox state to a more reduced state has also been proved to restore anti-oxidative activity and ligand binding activity of ALB impaired by oxidation [58]. It is quite notable serum ALB oxidation per se could be a factor that aggravates pathological status in liver diseases. The ALB fraction obtained from the serum of patients affected by severe alcoholic hepatitis, characterized by a prominent increase in HNA-2, strongly induced a respiratory burst of neutrophils obtained from healthy subjects [21]. Similar to this, HNA-1 (but not HNA-2), fractioned from the serums of healthy subjects, caused a “cytokine storm” in leucocytes obtained from health volunteers and cirrhotic patients [23]. Collectively, the oxidized shift of serum ALB redox state, initiated by oxidative stress in liver diseases, would not only impair physicochemical properties of ALB, but exacerbate vascular oxidative stress and inflammation through a neutrophil oxidative burst and a cytokine storm.

### 3.2. Renal Failures

Similar to liver diseases, serum ALB redox has been investigated well in the context of chronic kidney diseases (CKDs). Increases in HNA levels have been reported in these patients [11,15,19,32,42]. Most of these studies attended to the sum of oxidized ALB isoforms (i.e., HNA-1 + HNA-2), as determined by HPLC or colorimetric methods [15,19,32,65,66,67,68,69,70,71]. Still, some studies employed LC-MS/MS and indicated that an increase in cysteinylated ALB level was prominent in CKD patients [11,42]. The oxidized ALB levels paralleled the loss of renal function [32,67,68,69,70], and “oxidative” uremic toxin has been considered responsible for the ALB oxidation [34,72]. Hemodialysis treatment reversed the serum ALB redox state to a more reduced state [42,65]; reducing agents in dialysis membranes might be involved in the reversal, remaining an open question. As seen in studies of liver diseases, serum ALB obtained from CKD patients induced neutrophil oxidative burst [15,19]. As cardiovascular diseases (CVDs) are the common complications in CKD patients, oxidative stress aggravated by oxidized ALB-induced neutrophil respiratory burst might be responsible for the complication. Similarly, an ex vivo study by Pasterk et al. demonstrated that oxidized serum ALB isolated from end stage renal disease patients promoted platelet aggregation [20]. Magzal et al. demonstrated that serum ALB fraction obtained from hemodialysis patients facilitated the secretion of a pro-inflammatory cytokine IL-6 in human umbilical endothelial cells (HUVEC), and the secretion was attenuated by the reduction of ALB with dithiothreitol (DTT) [22]. Furthermore, according to the study Lim et al., HNA level was a positive predictor of CVD mortality in hemodialysis patients [73]. Thus, oxidative stress in CKD patients increases serum ALB oxidation, which would elicit neutrophil respiratory burst, platelet aggregation, and endothelial inflammation, thereby worsening the pathological conditions and complicating cardiovascular diseases. Finally, a recent study by Fujii et al., 2019 is notable as it demonstrated the association between serum ALB redox state and renal function in community-dwelling general population without CKD or its pretreatment [74], suggesting that serum ALB redox state would be even useful as an indicator of renal function in broad population.

### 3.3. Diabetes Mellitus

An increase in oxidized serum ALB level has also been observed in diabetes mellitus [11,19,32,75,76], but it has been less investigated compared with liver diseases and renal failures. Still, a recent study by Fukuhara et al. is notable as they demonstrated that serum HNA ratio was negatively correlated with the activities of daily living in diabetic older adults [76], suggesting that serum ALB redox would even reflect the quality of life in patients with diabetes mellitus.

### 3.4. Cardiovascular Diseases

The association between serum ALB redox state and CVDs have been discussed as complications of renal failures as discussed above [73]. Besides, Brioschi et al. reported significant increases in cysteinylation and homocysteinylation on serum ALB in heart failure patients compared with the health controls [13]. These authors further showed that the levels of these modifications were negatively correlated with oxygen consumption (VO_2_), an objective measure for the cardiopulmonary exercise capacity, indicating the potential association between serum ALB redox state and the severity of CVDs, as seen in the case of liver and kidney diseases. Notably, a recent study by Fujii et al., 2018 demonstrated the association between serum ALB redox state and the atherosclerotic indices of carotid intima-media thickness and plaque formation, despite the fact that only one third of the participants were diagnosed as hypertension [16].

### 3.5. Other Diseases

As discussed above, most of the studies on serum ALB redox state have been conducted in its association with major oxidative stress-related diseases such as liver diseases, renal failures, and diabetes mellitus. The following studies may therefore be outstanding because of their scientific uniquity. Bar-Or et al. found an increase in an cysteinylated plasma ALB in pregnant females diagnosed with fetal growth restriction (FGR) during pregnancy, compared with uncomplicated controls [38]. The authors viewed this as the manifestation of sustained oxidative stress resulting from ischemia, which is one of the etiological factors for FGR. A recent study by Inoue et al. investigated the role of ALB oxidation in cancer metastasis [24]. They revealed that serum ALB oxidation facilitated the generation neutrophil extracellular trap (NET) by neutrophil (termed NETosis) in cell assays and animal models, which promoted pulmonary cancer metastasis in animal models. These observations were further substantiated by a cohort study on patients with head and neck squamous cell carcinomas, reporting that patients with higher oxidized ALB levels had higher plasma NET levels as well as higher incidences pulmonary cancer metastasis. Another recent study by Ueno et al. showed that oxidized serum ALB increased in idiopathic and genetic Parkinson’s diseases [77]. These authors attributed this to accelerated aging-induced oxidative stress in these patients.

Biological fluids other than serum/plasma have also been applied to the analyses of the ALB redox state. Hayashi et al. examined the ALB redox state in aqueous humors of patients with senile cataract using HPLC [28], and it was found that ALB redox state was markedly shifted to a more oxidized state in the aqueous humors compared with their serums, their serums, implicating the local oxidative stress in the eyes. Boisvert et al. employed a LC-MS analysis to determine the post-translational modifications of ALB in amniotic fluids of pregnant female with gestational diabetes mellitus (GDM) [78]. It was found that the Cys residues in ALB were significantly cysteinylated or tended to be oxidized to sulfonic acid in pregnant female with GDM compared with non-GDM control, which can be viewed as a manifestation of systemic oxidative stress due to hyperglycemia in GDM patients. Costa et al. analyzed cerebrospinal fluids of Alzheimer’s diseases (AD) patients using HPLC and LC-MS, and found that both HNA-1 and HNA-2 were increased in cerebrospinal fluids as well as plasmas of AD patients compared with healthy controls [79]. Notably, a principal component analysis revealed that the difference between AD patients and healthy controls was more marked in cerebrospinal fluids than in plasma, implicating the involvement of oxidative stress in the brain region in the progression of AD.

Coronavirus disease 2019 (COVID-19) is an epidemic infection currently involving millions of people around the world, and some of the patients suffered from cytokine storm [80]. Based on the observations that oxidized shifts of serum ALB in various diseases are generally accompanied by decreases in serum ALB levels in various diseases, as well as a recent report that decreases in serum ALB level was frequently seen in many COVID-19 patients [81], Rahmani-Kukia et al. proposed the possibility that cytokine storm seen in COVID-19 patients could be attributed increased serum ALB oxidation [82], as seen in liver diseases [23]. While this decrease in ALB level is normally considered as acute inflammatory response, the above hypothesis may be worthy of being investigated and warrant clinical reports

## 4. Serum ALB Redox State in Response Physiological Conditions

Although most of the investigations on serum ALB redox state focused on its associations with various kinds of oxidative stress-related diseases as above, serum ALB redox state has also been affected by non-pathological factors such as aging and exercise. Furthermore, it has become evident that nutritional factors would modulate serum ALB redox state via ALB turnover rate. Serum ALB redox state in physiological conditions is summarized in Table 3.

### 4.1. Aging

Serum free thiols and protein thiols are converted to disulfides with aging [83]. Specifically, Era et al. reported the comparison of serum ALB redox state in young and old subjects (18–24 vs. 60–90 years of age) using HPLC as early as in 1995 [17]. It was found that HMA ratio was obviously higher in the old subjects compared with young subjects. Oettl et al. also presented in their review article that both HNA-1 and HNA-2 ratio increased with aging (ranging approx. 10–100 years of age) [10]. Similarly, Rossi et al. reported increases in cysteinylation and homocysteinylation of plasma proteins with aging in healthy subjects aged 20–93 [84]. Increases in HNA ratios with aging have been further confirmed in recent studies on patients and healthy controls [32,77]. The rationale for these observations has been attributed to oxidative stress accelerated by aging.

### 4.2. Exercise Training

As mentioned in previous review articles, the number of studies on the relationship between serum ALB redox state and exercise training has been limited as of today [2,10]. Imai et al. first reported oxidized shift of serum ALB redox state after exercise training (Kendo, a Japanese martial art fencing) in university elite athletes [18]. It was found that both HNA-1 and HNA-2 ratios increased significantly after the 5-day training camp compared with the baseline, indicating that serum ALB oxidation would be facilitated by reactive oxygen species generated by exercise training. This research group subsequently investigated the effect of a dietary antioxidant supplement, propolis (plant resins collected by honey bee), on oxidized shift of serum ALB redox state in an exercise program similar to the previous one [85]. Propolis intake during the exercise training alleviated the oxidized shift of serum ALB redox state after the exercise training, suggestion its effectiveness as a dietary antioxidant supplementation. Lamprecht et al. also investigated serum ALB redox state in Austrian policemen of a special anti-terrorism force after performing on a cycle ergometer at 70–80% VO_2max_ [86]. It was found that HNA-1, but not HNA-2, increased after the exercise training, and the increases were exercise-intensity dependent manner. This research group also examined the effects of dietary antioxidant supplementation, i.e., providing the subjects with encapsulated juice powder concentrate for 4–28 weeks before the exercise training [87]. However, this dietary supplementation had no effect on oxidized shift of serum ALB redox state after exercise training. A recent study by Ashikawa et al. is notable as they elucidated a positive correlation between serum HMA ratio and exercise capacity, as evaluated by 6-min walk distance, in women aged ≥75 years [88]. This observation would substantiate the association between oxidative stress and frailty [89].

### 4.3. Nutrition

As extensively discussed so far, serum ALB redox state has long been viewed as an oxidative stress marker. However, a series of recent animal studies have demonstrated that nutritional factors also modulate ALB redox state. Plasma ALB redox state shifted to a more oxidized state in rats fed a low-protein diet [25,26,90,91,92,93]. This shift was mediated by decreased ALB turnover including hepatic ALB synthesis rate [91], and was independent of oxidative stress as examined by plasma thiobarbituric acid reactive substance (TBARS) and advanced oxidation protein product (AOPP) concentration [26,91]. ALB redox state was also responsive to amino acid balance in diet [92,93]. Furthermore, plasma ALB redox state responded to a low-protein diet ingestion more sensitively compared with plasma ALB concentrations, a conventional protein nutrition biomarker [25,26]. Thus, ALB redox state would reflect the quality and quantity of dietary protein ingestion, and could be useful as a novel and sensitive protein nutrition biomarker. Clinical studies are essentially warranted to verify this notion.

## 5. Conclusions

**Figure 3:**
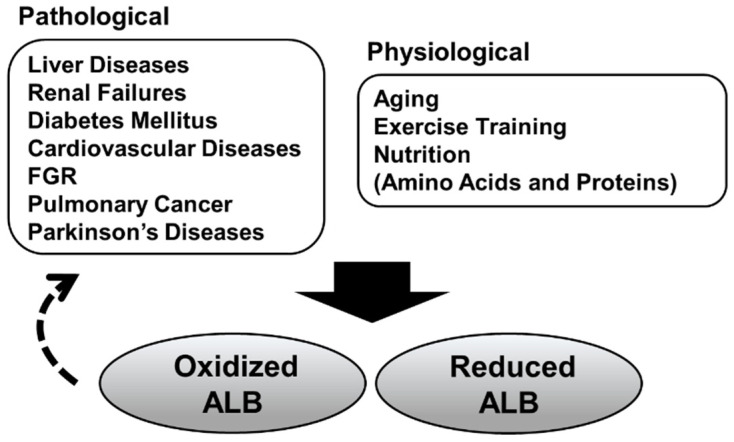
Pathological/physiological conditions associated with the serum albumin redox state. Increased oxidized albumin (ALB) has been observed in pathological and physiological conditions. Some recent studies have suggested that oxidized serum ALB isoforms per se could be involved in the progression of these diseases (dashed arrow). Furthermore, it has become evident that serum ALB redox state is also associated with protein nutritional status.

Serum ALB redox state is getting much more attention, and the biological interpretation of serum ALB redox state has thus changed significantly over the past decade (Figure 3). Specifically, the finding that oxidized serum ALB per se can be an aggravating factor for various diseases could lead to a conception for novel drug discovery. The usefulness of serum ALB redox state as a protein nutrition biomarker is not elucidated in humans, but is worthy of clinical investigation, especially targeting potentially protein-deficient generation such as older adults. Further progress of analytical techniques will support and boost the research field of serum ALB redox state.

## Figures and Tables

**Table 1:** Analytic methods for serum albumin (ALB) redox state.

Method	Strengths	Weaknesses
HPLC	Sperior to quantify ALB isoforms	Cannot determine post-translational modifications
LC-MS	Sperior to characterize post-translational modifications	Less quantitative
Colorimetric	Simple and rapid	Less accurate and informative

HPLC, high-performance liquid chromatography; and LC-MS, liquid chromatography coupled with mass spectrometry.

**Table 2:** Serum albumin (ALB) redox state in pathological conditions.

Condition	Method	Post-Translational Modification	References
Liver Diseases	LC-MS	Increase in Cys-, CysGly-, -SO_2_H, and -SO_3_H	[11,21,23,43]
	HPLC, Western blotting	Increase in HNA-1 and/or HNA-2	[35,58,59,60,61,63]
	HPLC	Decrease in HNA-1 and/or HNA-2 by BCAA supplementation	[58,62]
Renal Failures	LC-MS	Increase in Cys-	[11]
	HPLC, CD spectrum	Increas in HNA-1 and/or HNA-2	[15,32,65,66,67,70,71,73,74]
	LC-MS	Increase in Cys- and Glyc-	[42]
	HPLC	Decrease in HMA	[69]
Diabetes Mellitus	LC-MS	Increase in Cys-	[11]
	HPLC	Increase in HNA	[32,75,76]
Cardiovascular Diseases	HPLC	Increase in HNA	[16,73]
	LC-MS/MS	Increase in Cys- and HCys-	[13]
Fetal growth restriction	LC-MS	Increase in Cys-	[38]
Parkinson’s Diseases	HPLC	Increase in HNA	[77]
Alzheimer’s diseases (AD)	HPLC, LC-MS	Increase in HNA-1 and HNA-2 Increase in Cys- and -SO_2_H	[79]

CD, Circular Dichroism; Cys, cysteine; CysGly, cysteinylglycine; Glyc, glycocidation; HCys, homocysteine; HMA, human mercaptalbumin; and HNA, human non-mercaptalbumin.

**Table 3:** Serum albumin (ALB) redox state in physiological conditions.

Condition	Method	Post-translational Modification	References
Aging	HPLC	Increase in HNA-1 and/or HNA-2	[10,17,32,77]
	HPLC	Increase in Cys- and HCys- *	[83,84]
Exercise Training	HPLC	Increase in HNA-1 and/or HNA-2	[18,86,88]
Protein undernutrition	HPLC	Increase in HNA-1	[25,26,90,91,92,93]

* Thiols binding to total plasma proteins. Cys, cysteine; HCys, homocysteine; and HNA, human non-mercaptalbumin.
